# Complete genome and characteristics of cluster BC bacteriophage SoJo, isolated using *Streptomyces mirabilis* NRRL B-2400 in Columbia, MD

**DOI:** 10.1128/mra.00068-24

**Published:** 2024-02-23

**Authors:** Soven Verma Kumar, Nicholas Schaffer, Zainab Bharmal, Quinn Mood, 2022 UMBC Phage Hunters, Nameerah Abdullah, Ivan Erill, Steven M. Caruso

**Affiliations:** 1 Department of Biological Sciences, University of Maryland Baltimore County, Baltimore, Maryland, USA; 2 Departament d’Enginyeria de la Informació i de les Comunicacions, Universitat Autònoma de Barcelona, Bellaterra, Spain; Department of Biology, Queens College, CUNY, New York, New York, USA

**Keywords:** bacteriophage, *Streptomyces*, virus, genome, siphovirus, metal tolerance

## Abstract

Here, we present bacteriophage SoJo, a siphovirus infecting *Streptomyces mirabilis*, with a circularly permuted genome of 39 kbp and GC content of 71.5%. Its genome length and content are similar to that of other phages in the Actinobacteriophage Database BC cluster. SoJo was isolated from soil in Columbia, MD, USA.

## ANNOUNCEMENT

**Fig 1:**
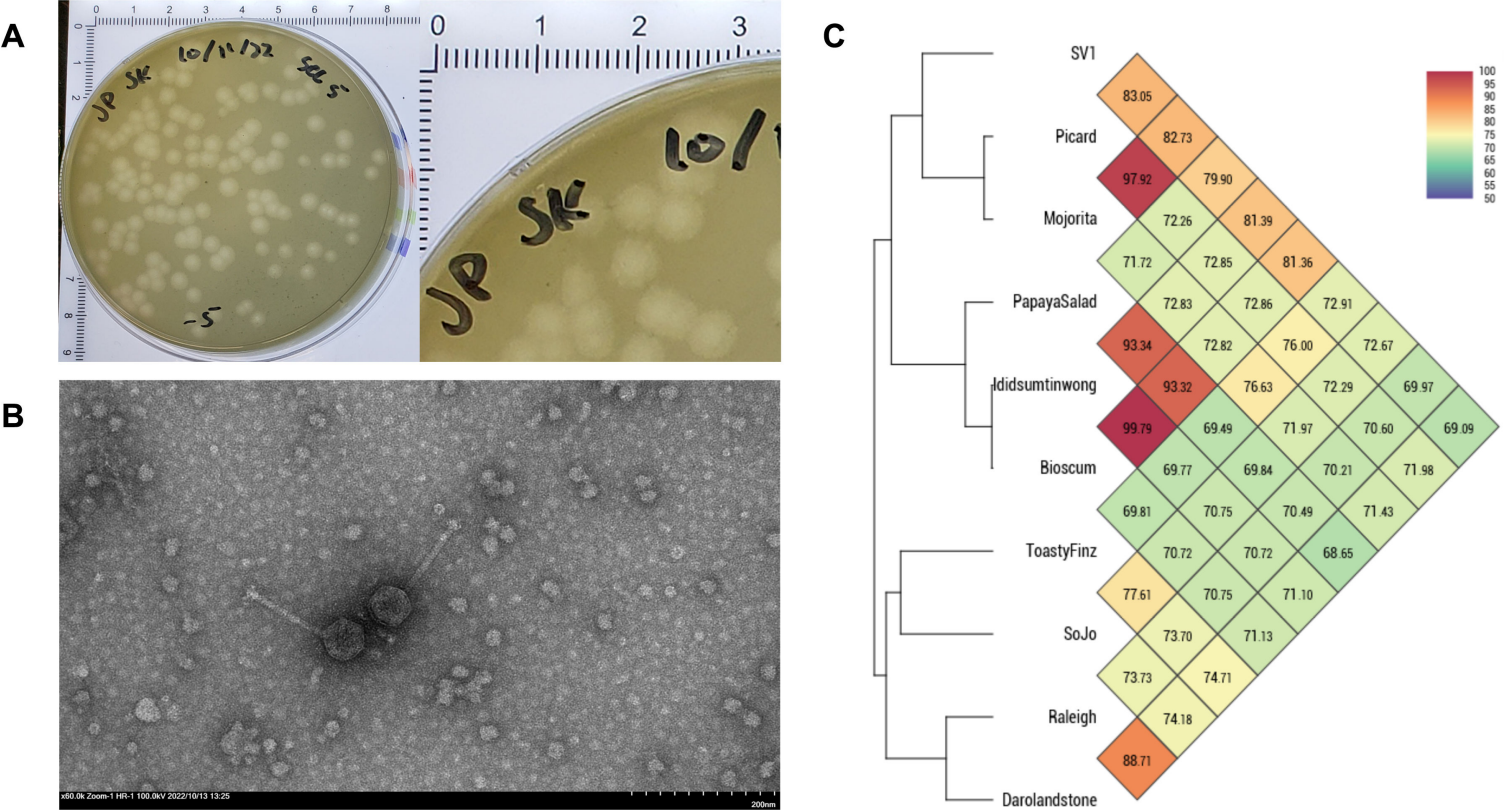
Phage imaging and estimation of genomic homology within the BC cluster. (**A**) Visible plaques present after 24 hours of incubation at 30°C. Plaques shown above were typically 3 mm in diameter and turbid. (**B**) Transmission electron micrograph of SoJo. Fresh plate lysate was negatively stained with 2% uranyl acetate and imaged using a Hitachi HT7800 120 kV TEM with an AMT Nanosprint15 B digital camera. Measurements of three different images of SoJo show an average capsid width of 55 nm, capsid length of 57 nm, tail length of 126 nm, and a tail width of 10 nm. (**C**) Average nucleotide identity as determined by OAT: OrthoANI v0.93.1 between SoJo and the nine other phages in the BC cluster. Higher percentages and darker colors correspond to more closely related phage genomes.

Bacteriophages are a prospective treatment option for bacterial infections that are difficult to treat with traditional antibiotics. Typically, lytic phages are studied for their potential efficacy in these cases, but studying lysogenic phages lends greater insight into bacterial pathogenicity via phage gene transfer (1). *Streptomyces* bacteriophage SoJo was directly isolated from warm (25°C), dry, and coarse dirt collected from Lake Elkhorn in Columbia, MD, USA (39.182121 N, 76.843525 W) using the bacterial host *Streptomyces mirabilis* NRRL B-2400. The soil sample was mixed with a phage buffer (10 mM Tris, 10 mM MgSO_4_, 68 mM NaCl, and 1 mM CaCl_2_) and centrifuged for 5 minutes at 2,500 rcf at room temperature. The supernatant was filtered (0.22 µm), combined with a 48 h culture of *S. mirabilis,* incubated for 10 minutes mixed with tryptic soy soft agar (BD), and plated on nutrient agar (BD Difco) supplemented with 10 mM MgCl_2_, 8 mM Ca(NO_3_)_2_, and 0.5% glucose. All methods were adapted from the Phage Discovery Guide (2). Following plating and overnight incubation at 30°C, turbid, round plaques with a diameter of approximately 2–3 mm were observed (Fig. 1A). A plate lysate was isolated after three rounds of plaque purification. Analysis via transmission electron microscope revealed SoJo’s siphoviral morphotype with a 126 nm long, flexible, non-contractile tail (Fig. 1B).

Phage DNA was isolated from fresh viral lysates using the Promega Wizard DNA preparation kit as described previously (3). Extracted DNA was sent to the Pittsburgh Bacteriophage Institute, where samples were prepared using the NEBNext Ultra II library preparation kit (NEB #E7645) and sequenced using Illumina MiSeq with v3 reagents and 150 bp single-end reads, yielding 2,380× coverage. Sequencing reads totaling 654,326 were assembled using Newbler v2.9 with default settings and checked for accuracy using Consed v29 as described previously (4–6). Genomic termini were determined as previously described (6). SoJo was determined to have a circularly permuted genome of 39,033 bp and a genomic GC content of 71.5%.

*Ab initio* gene prediction was accomplished using DNAMaster v5.23.6 (7) integrated predictive algorithms Glimmer v3.02b (8) and GeneMark v4.28 (9). Automatic gene calls were refined as per SEA-PHAGES annotation guidelines (2, 7). GeneMark.hmm version 2.5p (9) with Streptomyces_scabiei_87_22 as the selected species was used to further support start site calls. Functional annotations were generated manually using BLASTP (maximum *e-*value: 10^−7^) (10), Phamerator v3.0 (11), and HHPred v57v87 (minimum probability 90%) (12, 13). Default settings were used unless noted otherwise.

Annotation revealed that SoJo’s genome contains 53 genes including loci coding for predicted integrases, endonucleases, and recombinases, suggesting that SoJo is a temperate phage. Analysis by sequence similarity using OAT: OrthoANI v0.93.1 (14) revealed that SoJo is clustered within the BC1 subcluster of phages (15), which are also identified as temperate, and has an average nucleotide identity (ANI) of 72.26% with BC1 phages (Fig. 1C). Analysis of ANI values between BC clustered phages showed that SoJo is most similar to phage ToastyFinz (accession No. KY676784).

## Data Availability

SoJo is available at GenBank with accession No. OQ938586.1 and Sequence Read Archive (SRA) No. SRX20165783.
